# ‘In a dark place, we find ourselves’: light intensity in critical care units

**DOI:** 10.1186/s40635-017-0122-9

**Published:** 2017-02-07

**Authors:** Hannah J. Durrington, Richard Clark, Ruari Greer, Franck P. Martial, John Blaikley, Paul Dark, Robert J. Lucas, David W. Ray

**Affiliations:** 1 Division of Infection, Immunity and Respiratory Medicine, Faculty of Biology, Medicine and Health, AV Hill Building, University of Manchester, Manchester, M139PT UK; 2 Manchester Emergency and Intensive Care Research Group, Central Manchester Foundation Trust, Oxford Road, Manchester, M139WL UK; 3 Intensive Care Unit, Central Manchester Foundation Trust, Oxford Road, Manchester, M139WL UK; 4 Neuroscience and Experimental Psychology, Faculty of Biology, Medicine and Health, AV Hill Building, University of Manchester, Manchester, M139PT UK; 5 Greater Manchester Centre for Acute Tissue Injury and Trauma, Manchester Academic Health Science Centre, Division of Critical Care, Salford Royal NHS Foundation Trust, Greater Manchester, UK; 6 Division of Metabolism and Endocrinology, Faculty of Biology, Medicine and Health, AV Hill Building, University of Manchester, Manchester, M139PT UK

## Abstract

Intensive care units provide specialised care for critically ill patients around the clock. However, intensive care unit patients have disrupted circadian rhythms. Furthermore, disrupted circadian rhythms are associated with worse outcome. As light is the most powerful ‘re-setter’ of circadian rhythm, we measured light intensity on intensive care unit. Light intensity was low compared to daylight during the ‘day’; frequent bright light interruptions occurred over ‘night’. These findings are predicted to disrupt circadian rhythms and impair entrainment to external time. Bright lighting during daytime and black out masks at night might help maintain biological rhythms in critically ill patients and improve clinical outcomes.

## Background

ICU provides specialised care for critically ill patients around the clock; however, patients on ICU have disrupted circadian rhythms [1]. Disrupted circadian rhythms are associated with impaired survival from critical illness [2].

Circadian rhythm disturbances drive impairments in cognition (episodes of delirium) and alter the timing, duration and consolidation of sleep [3].

**Fig. 1:**
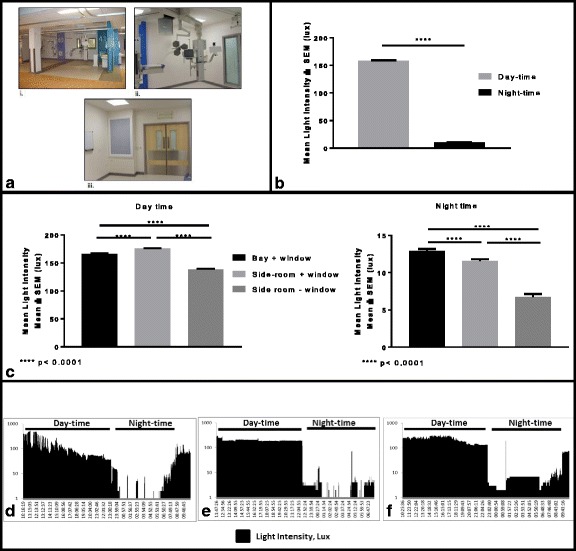
Artificial overhead lighting is present throughout the ICU and angle poise lighting is available on a stack at every bedside (**a**). A typical bed space in an open bay (bed absent). Note the window (*i*). A typical side-room with window (blind is down) (*ii*). A typical side-room without a window. Note the wall-mounted light box in place of a window (*iii*). Mean (±SEM) light intensity across ICU by day and night (**b**). Mean (±SEM) light intensity measured in different locations on ICU during the day and night (**c**). Fluctuating levels of light intensity are seen in an open bay bed space, which then gradually reduce over the day (**d**). Typical pattern of artificial lighting in a windowless side-room; a constant day-time light intensity of 180 lux, which abruptly reduces at night-time (**e**). A side-room with a window demonstrates a combination of natural and artificial lighting during the day (**f**). A low-intensity night-light has been left on for intervals overnight in (**e**) and (**f**). Overnight, there are several bright ‘pulses’ of light, indicating lights being switched on and off (**d**), (**e**) and (**f**)

Mechanical ventilation, sedation, severity of illness and the ICU environment (24-h light and noise) may all cause circadian disruption. It is now clear that light is the most powerful environmental influence on the circadian clock, acting through retinal photoreceptors [4]. Evidence suggests that disruption of a regular 24-h light-dark cycle increases morbidity and mortality [2]. It may be possible to ‘reset’ circadian rhythm in critically ill patients using bright light during the day and so improve clinical outcome. The therapeutic administration of morning bright light has been used for decades in psychiatry to treat seasonal affective disorder. Evidence from neonates suggests that cycled light improves sleep, alertness during the day, overall well-being and shortens time until discharge [5]. A longitudinal study in critical care implemented non-pharmacological environmental changes designed to reduce disturbing patients during the night (noise and light reduction by use of blackout masks) and demonstrated an impressive reduction in delirium and an improvement in sleep [6]. However, a recent clinical trial of continuous bright light therapy during the daytime in ICU (maximum light intensity 700 lux) concluded that there was no improvement in clinical outcome [7]. Current ICU guidelines recommend natural daylight for every patient room and artificial light that can be dialled up and down; currently, there is no indication as to what daytime light intensity should be achieved. The ICU at Central Manchester Foundation Trust opened in 2013 and employs windows and artificial, dial up and down, lighting systems. We measured light intensity in three different bed locations (a typical bay bed space, a side room with a window and a side room without a window Fig. 1a) within this newly built ICU, both during the day and night.

## Methods

Light intensity was measured in three different bed locations on ICU (a typical bay bed space, in a side room with a window and in a side room without a window Fig. 1a) over 24–48-h periods from August to October 2015. A small battery-operated light meter (made in house) was positioned at the patient’s eye level as close to the patient as possible and within 1 m. The light meter recorded ambient light intensity every 15 s. The light sensor of the light recorder was optimised for human vision (infrared filtered and peak sensitivity in the green part of the spectrum). No change in clinical practice was implemented.

Lighting on the ICU is provided by overhead ceiling lights for each bed space (Apollo Multicare, light intensity can be varied 150–500 lux). Overhead lights are switched on at 08:00 h and switched off at 23:00 h. Pendant operating lights are used for interventional procedures (Trumpf, light intensity up to 160,000 lux). Low-level lighting overnight is provided by Apollo Lunar.

## Statistics

Light intensity measurements were analysed as mean ± SEM across time and then by location of the light probe. An unpaired *t* test was used to compare the difference in means with significance *p* < 0.05.

## Results

Overall, the mean illuminance across all locations on ICU during the daytime was 158.9 ± 0.468 lux (mean ± SEM) and during the night-time was 10.44 ± 0.153 lux (mean ± SEM) Fig. 1b. Light intensity was then analysed by location of the probe on ICU. There was significantly increased light intensity during the daytime in the side-room with a window (175 ± 0.7614) compared to the open bay bed (166 ± 0.9106) and the side room without a window (138.8 ± 0.5933) Fig. 1c. Over night-time there was significantly lower light intensity in the side-room without a window (6.768 ± 0.3589) compared to the open bay bed (12.95 ± 0.2455) and the side-room with a window (11.6 ± 0.2278) Fig. 1c.

Distinct patterns of lighting in different locations on ICU can be recognised. Light recordings made in the open bay with window demonstrate gradual increases and decreases in light intensity at dawn and at dusk Fig. 1d. Light intensity within the windowless side-room reflects a typical pattern of artificial lighting: a constant ‘block’ of daytime light intensity at 180 lux, which abruptly reduces at ‘lights-off’, around 23:00 h Fig. 1e. Overnight, there are several bright ‘pulses’ of light (up to 300 lux), indicating lights being switched on and off as a procedure or check is being carried out Fig. 1e and f.

## Discussion

We present a description of 24-h profiles of light intensity to which patients in a typical ICU are exposed. Two important features of the ICU environment are apparent in our data: significantly low light intensities during the day and frequent overnight light interruptions.

Starlight on a clear night has an illuminance of ~0.001 lux and moonlight ~0.2 lux . In comparison, sunlight may be as intense as 100,000 lux, and on a heavily overcast, day will still be ∼ 1000 lux. A light intensity of 1000 lux is enough to entrain circadian rhythm [8]. The mean daytime light intensity measured across ICU in this study was low in comparison to natural daylight at 158.9 ± 0.468 lux (mean ± SEM). This is between 10 and 1000 dimmer than daylight. The consequence for circadian rhythms is hard to predict, but in the absence of scheduled sleep/darkness/activities (as occurs in ICU), an imposed 12-h light 12-h dark (dim light) cycle with domestic light at a similar intensity (200 lux) has been reported to be insufficient to maintain circadian phase position with some individuals drifting to later or earlier times relative to clock time [9]. A recent randomised controlled trial of continuous bright light therapy in ICU [7] failed to find an effect on the incidence of delirium. However, the maximal light intensity they achieved for the intervention was 700 lux (substantially below daylight levels) which may not have been a sufficiently large increase to observe an effect.

For bright light therapy to be effective in entraining circadian rhythm, it does not need to be continuous; in fact, exposure to three consecutive bright light ‘pulses’ for just 15 min can be more effective than continuous bright light [10]. This may also be more practical for use on ICU. Interestingly, despite the fact that many patients on ICU have their eyes closed, it is well recognised that bright light can entrain circadian rhythm through non-visual pathways via the retina [4].

Bright light at night can shift the phase of circadian rhythms and reduce their amplitude [11]. Such high-intensity light interruptions are necessary for the 24-h care given to critically ill patients; however, wearing a black out mask overnight might minimise this potential disruption to circadian rhythm.

In conclusion, this study has demonstrated significantly low light intensities during the day on a modern ICU, with frequent overnight light interruptions. With no clear guidelines as to what daytime light levels should be on ICU, a definitive clinical trial of bright light therapy (>1000 lux) during the day and black out masks at night is required to determine if there is a positive clinical outcome and also if circadian rhythms are re-established.

## Abbreviations

GAfREC: Governance Arrangements for Research Ethics Committees
ICU: Intensive care unit
